# Implementing an institution-wide electronic lab notebook initiative

**DOI:** 10.5195/jmla.2022.1407

**Published:** 2022-04-01

**Authors:** Erin D. Foster, Elizabeth C. Whipple, Gabriel R. Rios

**Affiliations:** 1 edfoster@berkeley.edu, Research Data Management (RDM) Program Service Lead (Research IT / UC Berkeley Library), University of California Berkeley, Berkeley, CA; 2 ewhipple@iu.edu, Assistant Director for Research and Translational Sciences, Ruth Lilly Medical Library, Indiana University School of Medicine, Indianapolis, IN; 3 grrios@iu.edu, Director, Ruth Lilly Medical Library, Indiana University School of Medicine, Indianapolis, IN

**Keywords:** electronic lab notebook, institutional collaborations, information management

## Abstract

**Background:**

To strengthen institutional research data management practices, the Indiana University School of Medicine (IUSM) licensed an electronic lab notebook (ELN) to improve the organization, security, and shareability of information and data generated by the school's researchers. The Ruth Lilly Medical Library led implementation on behalf of the IUSM's Office of Research Affairs.

**Case Presentation:**

This article describes the pilot and full-scale implementation of an ELN at IUSM. The initial pilot of the ELN in late 2018 involved fifteen research labs with access expanded in 2019 to all academic medical school constituents. The Ruth Lilly Medical Library supports researchers using the electronic lab notebook by (1) delivering trainings that cover strategies for adopting an ELN and a hands-on demo of the licensed ELN, (2) providing one-on-one consults with research labs or groups as needed, and (3) developing best practice guidance and template notebooks to assist in adoption of the ELN. The library also communicates availability of the ELN to faculty, students, and staff through presentations delivered at department meetings and write-ups in the institution's newsletter as appropriate.

**Conclusion:**

As of August 2021, there are 829 users at IUSM. Ongoing challenges include determining what support to offer beyond the existing training, sustaining adoption of the ELN within research labs, and defining “successful” adoption at the institution level. By leading the development of this service, the library is more strongly integrated and visible in the research activities of the institution, particularly as related to information and data management.

## BACKGROUND

Research institutions are currently facing a variety of information and data management challenges, such as increasing amounts of data regenerated, need for data storage, replicability and reproducibility, funder mandates for data sharing, and research misconduct. Within university settings, electronic lab notebooks (ELNs) are marketed as an improvement to paper notebooks, including improved information retrieval and data sharing [1–5]. Additionally, ELNs can ingest and store large(r) amounts of data, with many also offering integrated options for data backup and data security [6]. Lastly, ELNs may promote research reproducibility by providing improved documentation of research processes, particularly through features such as versioning and revision tracking [6, 7, 8].

Despite these purported advantages, numerous barriers may discourage researchers from transitioning from paper notebooks to ELNs. One primary challenge is cost; unless these tools are licensed by their home institutions, research labs or groups find themselves shouldering the cost of a license for these products. For some, this is a possibility via research funds; however, most find themselves unable or unwilling to cover such a cost [3, 7, 8, 9]. While many ELN products offer a “freemium” model whereby a given number of people or a given amount of storage is free, the limitations of the free versions rarely meet the needs of researchers over time. Additionally, features like institutional single sign-on and integration with an institution's cloud storage subscriptions (e.g., Google Drive, Box, Microsoft OneDrive) are often only available with institutional licensing. This is particularly notable for providing additional data security measures important to protect research information, as well as enable retention of this information and data by academic institutions.

Due to research universities' growing concerns over institutional cybersecurity and protecting data assets, universities increasingly require their researchers to ensure the security of their research data. This emphasis on data security, which includes guidance on entering, storing, and retaining data with third parties, can further complicate ELN adoption since these products often process and store data in the cloud [6, 10]. This highlights the importance of auditing an ELN product to ensure that third party companies' data practices are compliant with university standards. This step is easily overlooked when individual research labs or groups purchase licenses for ELN use outside of institutional units (e.g., Procurement Office) or use the free version of an ELN for academic research. Communicating the importance of the institutional security review and its function to protect research data and information long term—for both researchers and the institution—is crucial, as is ensuring that the security review process itself is well documented and timely in its completion.

The learning curves posed by integrating new software into researcher workflows create additional barriers to widespread ELN adoption. For researchers accustomed to paper notebooks, taking the time to implement and learn a new method of doing something as fundamental as recording and entering data may seem daunting. As with any tool, different tools and user interface learning curves contribute to the difficulty of adopting use of ELNs consistently across research labs or groups. As Argento notes: “One challenge that remains for the success of data management is the slow adoption of the ELN by research staff, partly because the ELN is still a work in progress, partly because old working habits are slow to change” [10]. ELNs are promoted as improving efficiency in access to research data, data management, communication, and information sharing, but often these efficiencies are not immediately evident, especially if the adoption of an ELN within a research group or lab is not thoughtfully and systematically approached.

Many researchers expect that ELNs must prove easier than using paper in order to justify changing an established workflow. However, an evaluation that only views ELNs through their functions for recordkeeping is incomplete, as ELNs offer functionality beyond documenting experiments and observations. Others have addressed this point by suggesting that ELNs should be viewed as a supplement to print lab notebooks but more dynamic [8].

A last point made by Rudolphi and Goossen is that academia tends to be more decentralized and diverse, which is something that many ELNs don't take into account; also, IT in academia is diverse with many storage/operating systems [11]. While this will likely be an ongoing challenge with ELN adoption in academia, libraries may offer assistance, particularly when it comes to helping researchers integrate an ELN into their research groups or labs from a research workflow and institutional policy perspective. The role that libraries play in supporting ELNs within academia is not well documented, even less so when it comes to academic medical libraries. A 2018 scan by Sayre et al. of thirty-five top academic research institutions found that ten libraries out of the thirty-five institutions provided ELN support of some kind [12]. Support came in the forms of instruction and consultations, library (or nonlibrary) developed guides or web pages about a particular ELN product, or ELNs in general. For those libraries that support research data services, Sayre et al. noted that ELNs provide a way of expanding support to encompass other areas, such as intellectual property, licensing, and digital preservation [12].

This was a perspective also shared by Grynoch, who compared twenty-five academic institutional policies related to data retention and data transferal, specifically in how these policies referenced ELNs [13]. In particular, this paper mentioned how the University of Massachusetts Medical Library worked closely with university IT to develop language around ELN transferal, as well as a process for retaining ELNs when researchers depart the institution. This guidance is intended to clearly communicate how researchers can “take” a copy of their ELN and research data and information with them, while also ensuring the university retains the original. While this type of partnership with IT and development of policy and procedure is not inherently built into many library research data services, it is natural in many ways by balancing the user-facing interaction of the library with the technical ability and infrastructure support offered by IT. Libraries engaging with campus ELN efforts can play a large role in contributing to decision-making on campus infrastructure and policy as these areas are highly relevant to the adoption and use of an ELN at an institution.

In order to provide a solution to the information and data management challenges facing the institution and their researchers, the Indiana University School of Medicine (IUSM) adopted an ELN system. LabArchives was the ELN selected for an institutional license, chosen based on an assessment of six other major universities. The identified universities in the assessment support (or supported) institutional licenses of LabArchives. IUSM is the largest medical school in the United States and supports a large research enterprise across its nine campuses in Indiana. The ELN initiative is led by the Ruth Lilly Medical Library and its librarians, who serve all campuses of IUSM and are based in Indianapolis. The medical library was chosen to lead this initiative due to the presence of a library data services program and a data librarian position, as well as the library's role supporting researchers across all disciplines at IUSM.

## CASE PRESENTATION

### Pilot phase (September 2018–March 2019)

Upon identifying LabArchives for piloting, fifteen principal investigators (PIs) volunteered to test out LabArchives in their respective labs following a call for early adopters from the IUSM Office of Research Affairs. From September 2018 to March 2019, these labs were trained in the use of LabArchives and began the process of adopting the ELN within their labs. Initial trainings were held by the vendor (i.e., LabArchives) with the Ruth Lilly Medical Library staff taking over training beginning in October 2018.

A user survey to these early adopter groups was distributed in March 2019 (Supplement 1). The survey was anonymous and distributed to the fifteen labs (sixty-seven individual users) with twenty-one users fully completing the survey (31% response rate). Of the respondents, the majority (67%, n=14) used LabArchives 50% to 100% of the time as opposed to another notebook (e.g., paper, other ELN). Twenty of the respondents (95%) also indicated that they adopted specific information and data management practices in their use of LabArchives, including

- Templates for consistently documenting activities like experiments (Supplement 2)
- Shared protocols in LabArchives notebooks
- Adoption and establishments of naming conventions
- Use of widgets for routine entries (e.g., calculating amounts, etc.)

Lastly, sixteen respondents (73%) agreed that use of LabArchives improved their labs' efficiency.

### Launch (April 2019)

In April 2019, LabArchives was made available to the entire School of Medicine—faculty, students, and staff—across all nine regional campuses. As the largest medical school in the country, IUSM has a full time equivalent (FTE) of nearly 7,000 full-time, part-time, and volunteer faculty; 1,265 residents and fellows; and an average class size of 360 medical students. While much of the school's research takes place on the Indianapolis campus, active research occurs on all nine campuses; the Ruth Lilly Medical Library and its librarians serve all campuses of IUSM while being based in Indianapolis.

As in the pilot phase, the same user survey was distributed again in October 2019 to assess use of LabArchives as well as perceptions of impact. In its second round, the survey was distributed to 233 users of LabArchives who were affiliated with research labs; sixty users submitted complete responses (26% response rate). Some highlights included the following: Of the respondents, 50% (n=30) used LabArchives 50% to 100% of the time (as opposed to another notebook). Additionally, 70% (n=42) of respondents agreed that lab leadership (i.e., PIs, lab managers) encouraged use of LabArchives. Forty of the respondents (67%) indicated that they adopted specific information and data management practices in their use of LabArchives, such as implementing templates for documenting experiments, sharing protocols, or establishing naming conventions. Lastly, thirty-two respondents (53%) agreed that use of LabArchives improved their labs' efficiency

**Figure 1:**
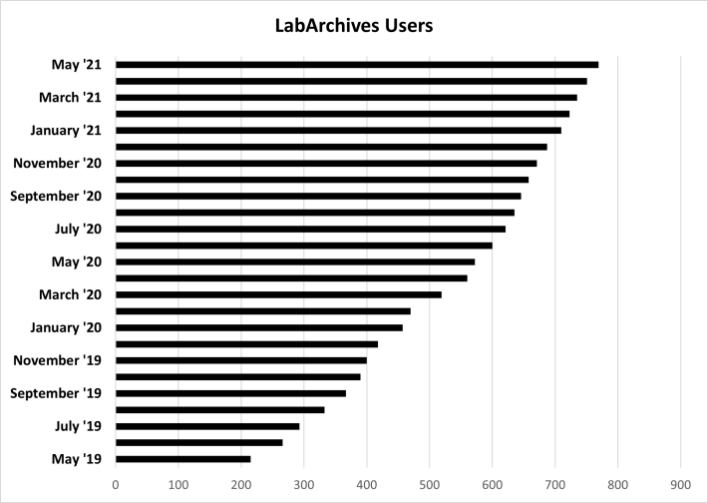
Number of LabArchives users May 2019–May 2021

Over the two years since the launch of LabArchives at IUSM, LabArchives user accounts have increased month over month (Figure 1). As of August 2021, there are 829 accounts across 22 (out of 25) IUSM departments. While there are individual users of LabArchives, most of the users are affiliated with a research group or lab. The continued adoption within IUSM is supported by monthly training sessions delivered through the Ruth Lilly Medical Library, individual consults, and email correspondence.

## DISCUSSION

### Broader impact of initiative

As the ELN pilot progressed, the data services librarian's role and time commitment increased and prompted the Ruth Lilly Medical Library's director to meet with IUSM Research Affairs leadership to negotiate FTE for the library's role. As a result of the negotiations, the medical library received funding for 1.0 FTE to focus on IUSM research initiatives, including the rollout of the ELN. Acquiring this extra position was a big win for the library and a commitment to long-term partnership between the medical library and the school's research office.

As the library assumed a leadership role for the ELN with support from IUSM Research Affairs, an Information Management Advisory Board (IMAB) was established with representatives from research affairs administration, marketing and communications, information technology, prominent PIs from the school, and the medical library. The IMAB was formed to guide the implementation and long-term support of the ELN, as well as other IUSM information and data management initiatives. Additionally, a representative from the medical library actively participates in a monthly Lab Optimization group focused on IUSM lab efficiency and lab space considerations, which often dovetails well with information management and ELN usage. Involvement with this group has led to a broader understanding of IUSM lab optimization priorities, alignment of mutual interests, and good information sharing. This group often works with faculty who may also be interested in help with data management and use of an ELN, which results in interested researchers being referred to the library.

The establishment of the IMAB in particular is a critical component to this initiative's success. The IMAB has given credence to the establishment and sustainability of the ELN initiative. IMAB members provided key feedback on the rollout and promotion of the ELN. Furthermore, PIs on the board have created models for other labs to follow. In addition to ELNs, the IMAB is examining information management issues (e.g., institutional data management plans) at IUSM on a much broader scale than lab notebooks. The board has allowed for important conversations related to policy and infrastructure development that may impact use of electronic lab notebooks, as well as other information and data management practices of researchers.

One important discussion held by the IMAB related to establishing data retention expectations for IUSM. As noted in the literature, the expectations around data retention are not often clear or detailed enough when it comes to *electronic* lab notebooks at institutions—existing guidance (if any) is geared toward paper lab notebooks [13]. This discussion led to the drafting of a data retention policy by campus library partners that includes minimum expectations for research data retention at IUSM, which explicitly mentions ELNs under the definition of “research data,” to be retained when PIs depart the school. This policy was approved July 1, 2021, and formally adopted on October 8, 2021 [14]. Moving forward, both IMAB and Lab Optimization members will be key groups to assist in raising awareness about this new policy and its expectations.

## LIMITATIONS

Responsibilities for supporting the ELN are currently divided among three librarians that directly support research at IUSM. In terms of FTE, this equates to roughly 1.5. While a significant impact of this work involved the gain of an entire (1.0) FTE, the unforeseen onset of the pandemic in March 2020 created challenges and delayed additional enhancements to ELN support at IUSM. For example, in 2020, the library anticipated more active promotion of LabArchives through planned outreach, such as conducting in-depth follow-ups with individual labs using LabArchives, adding additional seasonal training on new features, and targeted training of new staff brought onto research labs or groups. The pandemic and the transition of research at IUSM effectively put these plans on hold. Future plans include surveying IUSM LabArchives users again to inform current use practices and promotion activities.

Additionally, there is a need to identify metrics that accurately capture use and adoption of LabArchives. Currently, LabArchives provides metrics on users' “activities,” which are classified as anything from logging in and looking at notebook pages to actually adding information and data or making comments on notebook pages. These are a wide range of actions to group under a single metric category—having more granular metrics would better inform to what degree ELNs are utilized by users. For example, more visibility as to which users are solely logging in versus those who are logging in *and* adding content would assist with identifying outreach and support activities by the library to raise awareness on functionality and potential use of an ELN.

The question of adoption among IUSM LabArchives users continues to be discussed as well. To date, adoption has primarily been defined by increasing user numbers; however, those numbers do not tell the full story, namely because those users have varying degrees of engagement with LabArchives. Better integration of the LabArchives provided metrics and the user survey responses may assist with painting a richer picture of what “adoption” means at the school. Also, working with the IMAB and other campus partners to define what successful adoption of LabArchives looks like moving into the future would assist with these efforts.

### Changing a research culture

**Figure 2:**
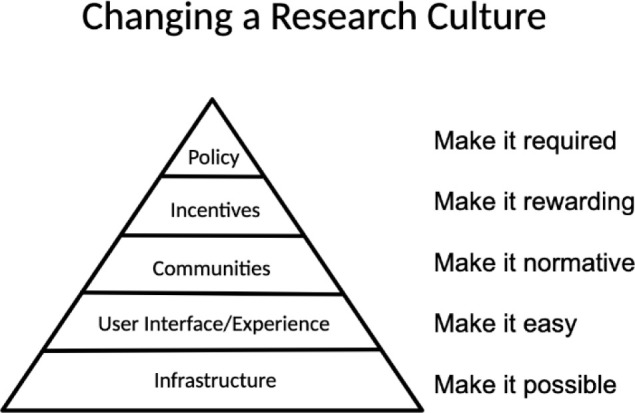
Strategy for culture and behavior change

Overall, implementation of the ELN initiative at IUSM has involved introducing changes to the research culture of the school. ELN products are tools that exist in a broader (research) ecosystem, and their use impacts not just the researchers but the broader administration of research at an institution. Brian Nosek, a researcher in open science, has identified five distinct steps institutions can take to begin to change a research culture, particularly if the interest is in retaining research data and enhancing the reproducibility of institutionally produced research [15]. The approach at IUSM (unwittingly) follows the steps outlined by Nosek in many ways (Figure 2).

- *Infrastructure* has been created by providing the centralized licensing of LabArchives by IUSM to allow for free access to affiliated researchers.
- *User Interface/Experience* is subjective in terms of ease of use; however, in the last user survey, the majority of respondents at IUSM reported that LabArchives made their work more efficient, which may correlate with “ease” of experience.
- *Communities* have been created through the early adopters of LabArchives at IUSM, which continue to grow each month. Use of ELNs may become normative naturally if adoption continues to rise. However, building communities will require additional effort, including reaching out to new faculty or creating spaces for IUSM users to showcase their use of LabArchives with peers. Beyond the researchers, the development of the IMAB and Lab Optimization groups have established another form of community between research faculty and staff; these spaces allow the school to address challenges that exist across areas of research.
- *Incentives* are an area of ongoing development: much of the incentivization so far has been centered around the improved efficiency of ELNs over paper notebooks. While responses from user surveys have supported that, it is important to more clearly elucidate what forms these efficiencies take in order to set expectations and incentivize users moving forward.
- *Policy* changes so far have been reflected in the changes to the institution's redefined expectations around research data retention. In the case of changing research culture, any policy that addresses ELN usage must ideally make clear how use of such a tool will result in a net benefit to the school, its researchers, and those that support the research enterprise.

Image by Brian Nosek, retrieved from: https://www.cos.io/blog/strategy-for-culture-change on October 8, 2021. Creative Commons License associated: https://creativecommons.org/licenses/by/4.0/

This initiative at IUSM has moved forward in many significant ways and—with the continued support from administration and engagement with researchers at IUSM—users (and use) of LabArchives are likely to steadily increase at the school. The success of the ELN initiative is just one piece of the research landscape at IUSM; this paper demonstrates the integral role libraries can have in supporting, growing, and shaping the information and data management priorities to move research reproducibility forward.

## Data Availability

Data associated with this article are available here: https://doi.org/10.7912/D2/29. Supplemental materials (Supplement 1: User survey form, Supplement 2: Template structure, Supplement 3: Class training slides/outline) can be found here: https://hdl.handle.net/1805/27739.
